# Advanced pulmonary tuberculosis in Alameda County: Ten-year incidence and risk factors

**DOI:** 10.1016/j.jctube.2024.100475

**Published:** 2024-08-05

**Authors:** Rachel Marusinec, Tessa Clifton, Amit S. Chitnis, Devan Jaganath

**Affiliations:** a Tuberculosis Section, Division of Communicable Disease Control and Prevention, Alameda County Health, Public Health Department, 1100 San Leandro Blvd, San Leandro, CA 94577, USA; b School of Public Health, University of California, Berkeley, 2121 Berkeley Way, Berkeley, CA 94704, USA; c Division of Pediatric Infectious Diseases, University of California, San Francisco, 550 16th St., 4th Floor, San Francisco, CA 94158, USA; d Center for Tuberculosis, University of California, San Francisco, 550 16th St., 3rd Floor, San Francisco, CA 94158, USA

**Keywords:** Tuberculosis, Epidemiology, Surveillance, United States, Delayed diagnosis

## Abstract

**Background:**

Advanced pulmonary tuberculosis (APT) may reflect delays in tuberculosis (TB) diagnosis and contribute to ongoing disease transmission and poor outcomes. We characterized trends and factors associated with APT over ten years in a high TB-burden county within the United States.

**Methods:**

We evaluated microbiologically and clinically confirmed TB cases reported to the Alameda County Public Health Department during 2010–2019. APT was defined as the presence of cavitation on chest imaging and positive acid-fast bacilli sputum smear. Over the ten-year period, we determined overall incidence and annual trends, and conducted multivariable logistic regression to identify sociodemographic and clinical factors associated with APT.

**Results:**

We included 997 cases with pulmonary TB, of which 128 (12.8 %) had APT. The 10-year incidence of APT was 8.8 cases per 100,000, with no significant change in proportion over time. The median age of cases with APT were 50 years (IQR 32–61), 68 % were male, and 78.9 % were non-US-born. On multivariable assessment, APT cases were more likely than non-APT cases to use drugs in the past year (aOR 2.43, 95 % CI 1.10–35.09), to have diabetes (aOR 2.51, 95 % CI: 1.59–3.96), and be HIV negative (aOR 9.32 versus HIV positive, 95 % CI 1.87–169.77). While US nativity was not significantly associated with APT, it was an effect modifier. In stratified analysis, APT was more likely among those with drug-use in the last year among US-born, while diabetes was associated with APT among non-US-born individuals.

**Conclusions:**

APT remains a substantial proportion of TB cases. Efforts to increase awareness and access to care are needed for key risk groups including those with recent drug use or diabetes, with consideration of cultural and linguistic factors given differences by US nativity.

## Highlights

• The proportion of advanced pulmonary tuberculosis (APT) did not decline and remained stable in Alameda County from 2010-2019.
• Diabetes and recent drug use were associated with APT; further interventions are needed to support earlier access to care.
• Risk factors for APT differed by US-nativity, and public health efforts should be tailored to cultural and linguistic needs.

## 1 Introduction

Although tuberculosis (TB) incidence rates declined in the United States (US) during 1993–2019 [1], TB elimination has not been achieved and TB still contributes to significant health disparities, with highest burden among non-US-born individuals and those with a lower SES background [2], [3]. While TB incidence rates have decreased, the proportion of individuals with advanced pulmonary tuberculosis (APT), defined as pulmonary TB with the presence of cavitation on chest radiograph and a positive sputum acid-fast bacilli smear result, has increased during 1993–2013 [4], [5]. Individuals with APT contribute to ongoing TB transmission and are at higher risk of poor outcomes including prolonged treatment with adverse events, post-TB lung disease, and death [6], [7].

APT is a late presentation of TB and has been used as a proxy measure for delayed diagnosis. Risk factors for APT in the US have included alcohol use, younger age, and multidrug- resistant (MDR) TB [4], [5], but these factors have been assessed primarily in low TB incidence settings. Greater characterization of APT cases is needed to guide clinical and public health interventions for earlier TB detection and treatment initiation. These interventions targeted toward earlier detection and treatment of APT cases may in turn reduce poor TB outcomes and ongoing disease transmission.

Alameda County is a high TB-burden county in Northern California, with an average yearly TB incidence of 8.1 cases per 100,000 population. In this setting, we examined incidence trends and social and epidemiological factors associated with APT during 2010–2019.

## 2 Methods

### 2.1 Setting

Alameda County Public Health Department serves over 1.5 million individuals in Alameda County, excluding the City of Berkeley, which is its own local health jurisdiction. One-third of the county’s residents are non-US-born; 22 % of the population are Hispanic/Latino, 11 % are African American/Black, 35 % are Asian, and 29 % are non-Hispanic White [8]. During 2010–2019, yearly TB incidence rates in Alameda County ranged from 7.4–12.6 cases per 100,000 population.

### 2.2 Data

**Fig. 1:**
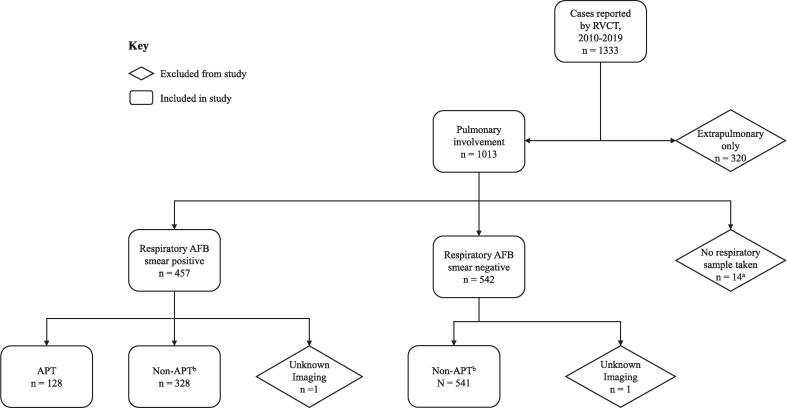
Flowchart of pulmonary tuberculosis case classification, Alameda County, 2010–2019. Abbreviations: AFB: acid-fast bacilli; APT, Advanced pulmonary tuberculosis; RVCT, Report of a Verified Case of Tuberculosis; ^a^Non-respiratory AFB smear taken, n = 8; AFB smear not done, n = 6. ^b^Characteristics of APT defined as the presence of cavitation on chest radiograph, and a positive acid-fast bacilli sputum smear result. One characteristic of APT was defined as having either cavitation on chest radiograph or a positive acid-fast bacilli sputum smear result. For the purpose of these analyses, non-APT (n=869) included those with no features of APT (n=499) and one feature of APT (n=370).

We included microbiologically and clinically confirmed TB cases meeting the CDC surveillance case definition [9] that were reported during 2010–2019 to the Alameda County Public Health Department. Reported cases were investigated by public health staff and data were sent to the California Department of Public Health using the Report of Verified Case of Tuberculosis (RVCT) form through the California Reportable Disease Information Exchange (CalREDIE). RVCT data was pulled from CalREDIE for this analysis and de-identified. Cases were excluded from the analysis if there was extrapulmonary involvement only, or absence of a respiratory sample or no imaging data (Fig. 1).

Because this analysis of TB surveillance and other routinely collected public health data were conducted to enable ACPHD to monitor, assess, and inform local TB public health interventions, no human subject review was required.

### 2.3 Definitions

Tuberculosis cases were confirmed according to the CDC case definition as a case that meets the clinical case definition or is laboratory confirmed [9]. APT was defined as having the following characteristics: presence of cavitation on chest radiograph; and a positive acid-fast bacilli sputum smear result [4]. Cases with neither or only one characteristic of APT were considered non-APT for this analysis. Cases were considered multidrug-resistant if cultures were resistant to both isoniazid and rifampin. For this analysis, drug usage was defined as use of either injection or non-injection drugs in the 12 months prior to TB diagnosis. An individual was considered unemployed/not seeking employment if they were 18 years of age or older, and their occupation was listed as either unemployed or not seeking employment on the RVCT.

The Healthy Places Index (HPI) is a geographical index measure representing social conditions that affect health [10] and was used as a proxy for socioeconomic status and health-related environmental conditions. Index values for Alameda County census tracks were assigned quartiles, with Quartile 1 representing the census tracts with social conditions least conducive to health and Quartile 4 representing census tracks with conditions most conducive to health.

### 2.4 Analysis

We calculated the incidence rate of APT per 100,000 population, with the denominator based on Esri population estimates. Trends in annual proportion of APT cases, overall and by nativity, were assessed using linear weighted regression, with significance defined as p-value < 0.05. To determine the association of demographic and clinical variables with APT, we compared APT and non-APT cases using descriptive statistics, including chi-square tests and Fisher’s Exact Test for categorical variables, and the mood median test for continuous variables. Univariate regression models were also used to measure association between specific clinical and demographic variables and APT. Variables with p < 0.2 in univariate analyses and a priori identified risk factors based on the published literature (i.e., age, sex at birth, race/ethnicity, nativity, unemployment, and TB contact) were included in a stepwise logistic regression model. The likelihood ratio test was used for model comparison, and the Hosmer–Lemeshow test was used to assess goodness-of-fit for the final model. Significance for the model was defined as a 95 % confidence interval (CI) of odds ratios (ORs) that did not cross one, and descriptive statistics were considered significant at p < 0.05. Data were analyzed using R version 4.0.2 with the packages: *stats, coin, questionr, sfsmisc, lmtest,* and *ResourceSelection*.

## 3 Results

### 3.1 Incidence of APT

**Fig. 2:**
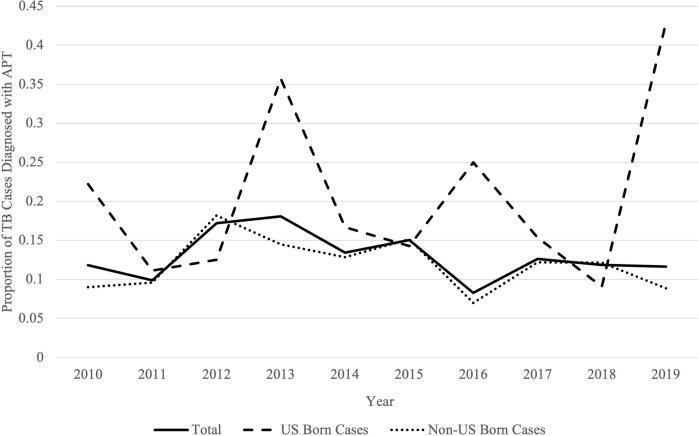
Trends^a^ in proportion of pulmonary tuberculosis cases with advanced pulmonary tuberculosis, by year and nativity, Alameda County, 2010–2019. Abbreviation: APT, Advanced pulmonary tuberculosis. ^a^All trends, p > 0.05.

During 2010–2019, 1,333 TB cases were reported to Alameda County; a total of 997 cases were included for this analysis (Fig. 1). APT was detected in 128 cases, comprising 12.8 % of all reported pulmonary TB cases within the analysis period. Of non-APT cases, one characteristic (i.e., either cavity on chest radiography or acid-fast bacilli sputum smear positive) was detected in 370 of 869 (43 %) individuals. The 10-year incidence of APT in Alameda County was 8.8 cases per 100,000 population. There was no significant change in the proportional trend of APT cases over time (p = 0.52), including when stratifying by country of nativity (US-Born: p = 0.49, non-US-Born: p = 0.62, Fig. 2).

### 3.2 Characteristics of APT

The median age among individuals with APT was significantly lower than those without APT (50 years versus 55 years of age, p = <0.01; Table 1). Hispanic and non-Hispanic Black individuals made up a significantly higher proportion of APT cases than non-APT cases, respectively (19.5 % vs 11.0 %, p < 0.01, and 13.3 % vs 7.5 %, p < 0.01). A total of 856 (86 %) pulmonary TB cases were non-US-born, though US-born persons were significantly more likely to have APT compared to non-US-born persons (21.1 % vs 13.0 %; p = 0.02). Less than half of individuals with APT were unemployed or not seeking employment (n = 57, 44.5 %), while greater than half of individuals without APT were unemployed or not seeking employment (n = 476, 54.8 %). Drug use was significantly higher among those with APT than those without APT (n = 15, 11.7 % vs n = 35, 4.0 %; p < 0.01). Patients with APT, compared to without APT, were also significantly more likely to have diabetes (n = 48, 37.5 % vs n = 202, 23.2 %; p < 0.01), and have symptoms as their primary reason for TB evaluation (n = 113, 88.3 % vs n = 610, 70.2 %, p < 0.01). Positive HIV status was significantly lower among patients with APT compared to without APT (n = 1, 0.8 % vs. n = 34, 3.9 %, p < 0.01). A significantly higher percentage of patients with APT, compared to without, completed treatment through directly observed therapy (n = 96, 75 % vs. n = 492, 56.5 %, p < 0.01).

**Table 1:** Comparison of demographic and clinical characteristics among pulmonary tuberculosis case-patients, by burden of disease, Alameda County, 2010–2019.

**Characteristic**	**No APT** **(n = 869)**	**APT** **(N=128)**	***P*-value**^a^
***Demographic***			
Age, median (IQR)	55 (34–72)	50 (32–61)	< 0.01
Age category, n (%)			0.02
0–24y	102 (11.7)	18 (14.1)	
25–44y	196 (22.6)	34 (26.6)	
45–64y	254 (29.2)	47 (36.7)	
≥65y	317 (36.5)	29 (22.7)	
Sex at birth, male, n (%)	527 (60.6)	87 (68.0)	0.14
Ethnicity, n (%)			< 0.01
Hispanic	96 (11.0)	25 (19.5)	
Non-Hispanic			
White	54 (6.2)	9 (7.0)	
Black	65 (7.5)	17 (13.3)	
Asian	635 (73.1)	73 (57.0)	
Other	19 (2.2)	4 (3.1)	
Country of birth, n (%)^b^			0.02
US-born	113 (13.0)	27 (21.1)	
Non-US-born	755 (86.9)	101 (78.9)	
*TB social risk factors, n (%)*			
Homeless in the year prior to diagnosis	18 (2.1)	2 (1.6)	1
Correctional facility resident at diagnosis	11 (1.3)	4 (3.1)	0.11
Unemployed/not seeking employment^c^	476 (54.8)	57 (44.5)	0.04
Substance misuse			
Excess alcohol use in past year	40 (4.6)	11 (8.6)	0.08
Drug^d^ use in past year	35 (4.0)	15 (11.7)	< 0.01
Injection drugs	4 (11.4)	0 (0.0)	
Non-injection drugs	33 (94.3)	15 (100)	
*TB clinical risk factors, n (%)*			
Diabetes	202 (23.2)	48 (37.5)	< 0.01
End stage renal disease	22 (2.5)	1 (0.8)	0.34
Immunosuppression, not HIV	61 (7.0)	4 (3.1)	0.12
Contact of recent TB case	38 (4.4)	5 (3.9)	1
Alameda County Healthy Places Index Quartiles, n (%)^e^			0.64
1st Quartile	259 (29.8)	42 (32.8)	
2nd Quartile	255 (29.3)	40 (31.3)	
3rd Quartile	178 (20.5)	24 (18.8)	
4th Quartile	120 (13.8)	13 (10.2)	
Unknown	57 (6.6)	9 (7.0)	
Primary reason for TB evaluation, n (%)			< 0.01
Symptoms	610 (70.2)	113 (88.3)	
Incidental abnormal chest x-ray	157 (18.1)	9 (7.0)	
Contact investigation	25 (2.9)	2 (1.6)	
Other	77 (8.9)	4 (3.1)	
*Clinical, n (%)*			
HIV status, n (%)			< 0.01
Positive	34 (3.9)	1 (0.8)	
Negative	597 (68.7)	104 (81.3)	
Unknown	238 (27.4)	23 (18.0)	
Drug resistance, n (%)^f^			
Susceptible to first-line TB therapy^g^	582 (86.5)	102 (82.3)	0.21
Isoniazid monoresistant	65 (9.7)	17 (13.7)	0.20
Pyrazinamide monoresistant	12 (1.8)	2 (1.6)	1
Multidrug resistant^h^	10 (1.5)	3 (2.4)	0.44
Radiology, n (%)			
Miliary findings on X-ray	16 (1.8)	1 (0.8)	0.71
Miliary findings on CT	31 (3.6)	5 (3.9)	0.80
TB treatment type, n (%)			< 0.01
Directly Observed Therapy	492 (56.6)	96 (75.0)	
Self-Observation	165 (19.0)	5 (3.9)	
Both	191 (22.0)	26 (20.3)	
Unknown	21 (2.4)	1 (0.8)	
Dead at Diagnosis	12 (1.4)	0 (0 %)	0.38
Died during therapy, n (%)	67 (7.7)	13 (10.2)	0.44
Died in first 8 weeks of treatment	30 (44.8)	5 (38.5)	0.75

Abbreviations: APT, advanced pulmonary tuberculosis; CT, Computed Tomography; HIV, Human Immunodeficiency Virus; IQR, Interquartile Range; TB, Tuberculosis.

a p-value calculated using chi-square, Fisher’s test, or Mood’s median test.

b Excludes one case-patient with an unknown country of birth.

c Does not include minors and retirees. Retirees were defined in RVCT and minors were defined as individuals under the age of 18.

d Drugs include injectable and non-injectable drugs, excluding tobacco and alcohol.

e Based on Healthy Places Index Score, which is a measure of social conditions that influence a communities health [10].

f Analyses restricted to case-patients with drug susceptibility results; APT, n = 124; No APT, n = 673.

g First-line TB therapy includes: isoniazid, rifampin, pyrazinamide, ethambutol.

h Multidrug resistant is defined as phenotypic drug resistance to both rifampin and isoniazid.

### 3.3 Multivariable risk factors associated with APT

Adjusted OR (aOR) were calculated for APT and multiple demographic, clinical, and social variables, including race/ethnicity, sex at birth, age, US nativity, drug use, diabetes, TB infectious contact status, HIV status, and employment status. As shown in Table 2, the aOR of APT among those that used drugs in the year prior to TB diagnosis were 2.43 (95 % CI: 1.13–5.09) times higher than the odds of APT among non-drug users (Table 2). The odds of APT were also significantly higher among those with diabetes than those without (aOR 2.51, 95 % CI: 1.59–3.96). Individuals who tested negative for HIV had 9.32 times higher odds of having APT (95 % CI: 1.87–169.77) than persons living with HIV (PLWH); individuals with no HIV test results had 5.91 times higher odds of having APT (95 % CI: 1.12–109.53) than PLWH.

**Table 2:** Factors associated with advanced pulmonary tuberculosis, Alameda County, 2010–2019.

**Variable**	**Adjusted Odds Ratio**^a^ **(95 % Confidence Interval)**
Age Category	
0–24 y	1.12 (0.57–2.16)
25–44 y	Ref.
45–64 y	0.88 (0.52 –1.50)
65+ y	0.54 (0.29–1.01)
Ethnicity	
Hispanic	1.13 (0.47–2.87)
Non-Hispanic	
White	Ref
Black	1.35 (0.53–3.55)
Asian	0.63 (0.30–1.48)
Other	0.83 (0.19–3.04)
Sex	
Male	Ref
Female	0.79 (0.52–1.20)
Country of Birth	
Not US Born	Ref
US Born	1.34 (0.73–2.41)
Drug use	
No	Ref
Yes	2.43 (1.13–5.09)
Diabetes	
No	Ref
Yes	2.51 (1.59–3.96)
Contact of Infectious TB Case	
No	Ref
Yes	0.70 (0.23–1.78)
HIV Status	
Positive	Ref
Negative	9.32 (1.87–169.77)
Unknown	5.91 (1.12–109.53)
Unemployed/Not Seeking Employment	
No	Ref
Yes	0.81 (0.52–1.25)

Abbreviations: APT, advanced pulmonary tuberculosis; HIV, Human Immunodeficiency Virus; TB, Tuberculosis.

a Multivariate model comprised of all variables listed in the table.

### 3.4 Risk factors for APT when stratified by US nativity

Although the multivariate model did not find a significant association between US nativity and APT, interaction was tested and found to be present between US nativity and race/ethnicity and US nativity and drug use. aORs were calculated and presented for the final model without interaction terms (Table 2) and stratified by US nativity (Table 3). A significant association between diabetes and APT was detected in non-US-born individuals (aOR: 2.55, 95 % CI: 1.56–4.22), while drug use in the last year was significantly associated with APT among US-born individuals (aOR: 12.17, 94 % CI: 3.00–60.87). There was significantly lower likelihood of APT among females and individuals aged > 65 years old in non-US-born persons, while APT was significantly lower in unemployed individuals/those not seeking employment for US-born persons. The association between race/ethnicity and APT was not significant when stratified by US nativity.

**Table 3:** Factors associated with advanced pulmonary tuberculosis, by nativity, Alameda County, 2010–2019.

	**Adjusted Odds Ratio for US Born** **(95 % Confidence Interval)** **N = 140**	**Adjusted Odds Ratio for Non-US Born** **(95 % Confidence Interval)** **N = 856**
Age Category		
0–24 y	0.64 (0.13–2.98)	1.38 (0.63–2.91)
25–44 y	Ref	Ref
45–64 y	2.48 (0.61–11.38)	0.74 (0.40–1.35)
65+ y	2.71 (0.41–19.38)	0.45 (0.23–0.90)
Ethnicity		
Hispanic	3.10 (0.42–25.82)	0.93 (0.33–2.90)
Non-Hispanic		
White	Ref	Ref
Black	2.84 (0.63–15.83)	0.54 (0.12–2.21)
Asian	2.31 (0.38–16.69)	0.45 (0.18–1.27)
Other	–a	1.51 (0.30–7.29)
Sex		
Male	Ref	Ref
Female	2.03 (0.73–5.92)	0.60 (0.36–0.98)
Drug use		
No	Ref	Ref
Yes	12.17 (3.00–60.87)	0.77 (0.17–2.57)
Diabetes		
No	Ref	Ref
Yes	2.28 (0.44–11.32)	2.55 (1.56–4.22)
Infectious TB Contact		
No	Ref	Ref
Yes	0.51 (0.06–2.79)	0.84 (0.19–2.66)
HIV Status		
Positive	Ref	Ref
Negative	–a	4.01 (0.77–73.90)
Unknown	–a	2.44 (0.43–46.08)
Unemployed/not seeking employment		
No	Ref	Ref
Yes	0.14 (0.03–0.58)	0.95 (0.58–1.55)

Abbreviations: APT, advanced pulmonary tuberculosis; HIV, Human Immunodeficiency Virus; TB, Tuberculosis.

a Group size too small for stable estimate.

## 4 Discussion

Delayed diagnosis of TB can lead to poor outcomes, including increased transmission and outbreaks, and longer treatment duration and high recurrence rates [6], [11]. APT has been used as a proxy for delayed TB diagnosis, [4], [5] and during 2010–2019, we noted that 12.8 % of pulmonary TB cases in Alameda County were APT. There was no trend in APT proportion over the ten-year period, and APT was more likely among persons living with diabetes and with recent drug use; however, this association varied by US birth status. In a high-burden TB county in the US, we thus found that APT remains an ongoing challenge, and that there are key groups that may benefit from interventions to support earlier TB evaluation and treatment.

Our estimate of APT proportion was lower than that previously estimated in the US (18.5 %–26.5 %) [4]. This follows observations by other studies that have noted incidence of APT and its characteristics to be higher in lower TB incidence areas compared to higher incidence areas [4], [12]. There was no apparent trend in APT proportion from 2010–2019 (Fig. 2), while other parts of the US have detected increases over time in the proportion of APT, cavitation, and positive acid-fast bacilli sputum smears [4], [5]. However, these studies have focused on culture-confirmed cases and examined trends during time periods that occurred prior to our analyses.

Diabetes and drug use were found to be associated with APT; however, upon stratification by nativity, drug use was found to be associated with APT only among US-born, and diabetes was found to be associated with APT among only non-US-born cases. These differences by country of birth likely reflect the greater incidence of risk factors in these populations, leading to delays in care and more advanced TB. A higher percentage of US-born TB patients has reported drug use compared to non-US-born TB patients [13], and pulmonary TB cases among drug users have been found to have higher proportions of acid fast bacilli smear positive sputum specimens and cavitation on chest radiography compared to non-drug users. Studies have found patient delay for TB care among persons who use drugs [14], and those with recent drug use are less likely to seek care and encounter barriers to care in general [15], [16].

Conversely, non-US-born individuals have a higher prevalence and odds of diabetes than US-born individuals [17], and diabetes is associated with a higher likelihood of cavitation [6]. In addition, rates of active TB disease have been found to be higher among non-US-born patients with diabetes than US-born patients with diabetes in California [18]. The literature on the direction of association of diabetes on TB diagnostic delay is varied, and this association may, in part, be related to access to the health system, awareness of the patient and provider of the diabetes diagnosis, and recognition by the provider that diabetes is a risk factor for TB [19], [20], [21].

In the overall model, PLWH were less likely to have APT. The limited number of cases prevented further characterization by US nativity. Other studies have found HIV positivity associated with lower odds of cavitation, and decreased prevalence among PLWH compared to non-HIV-infected individuals [4], [5]. While PLWH are more likely to have paucibacillary disease and thus less cavitation [22], PLWH also may be more likely to be diagnosed with TB earlier, as testing for latent TB infection is recommended for PLWH by the CDC [23]. This highlights the benefits of earlier or more frequent testing for groups at risk for TB.

We detected decreased odds of APT for those 65 years and older compared to persons aged 25–44 years old among non-US-born patients. Other studies have also found adults ≥ 65 years old to be less likely to have cavitary disease than younger patients, and the proportion of APT to be lower among those ≥ 65 years old compared to lower age groups [4], [5], [24]. This may additionally reflect that recent immigrants are younger and may have reduced access to healthcare [25]. Among non-US-born patients, females were less likely to have APT than males. While some studies have noted lower proportions of cavitation and sputum-smear positivity among females compared to males, others have not detected significant associations between APT and sex [5], [26]. In contrast, males have been noted to have a higher prevalence of TB internationally [27]. Among US-born persons, the odds of APT among those unemployed or not seeking employment were 0.14 times those among persons who were retired or employed in the 12 months prior to TB diagnosis. Other studies have also found lower prevalence of APT among the unemployed, with increased proportion of APT in employed individuals over time [4].

The majority of TB cases in Alameda County were in the lowest two HPI quartiles (Table 1). However, no association was detected between APT and HPI (Table 1). While overall higher rates of TB have been noted to be associated with lower SES status [2], [3], other studies have also detected no association of area-level SES and APT features [28]. It could be that individual-level risk factors for APT are stronger than ecological SES factors among the Alameda County population. In addition, though SES and race and ethnicity are known risk factors of TB in our county and the US, our analysis did not detect increased odds of APT by race and ethnicity, although the distribution of overall TB cases varied significantly by race and ethnicity (Table 1). While being a contact of an infectious TB case was associated with lower likelihood of APT, it was not statistically significant. TB contacts may be screened and treated for TB disease earlier and this may lead to reduced risk of APT; however, there are challenges to identifying and screening all contacts and individuals with pulmonary TB disease who may present to care first with symptoms or abnormal imaging [29], [30].

Our findings reflect 10 years of surveillance data in a high TB-burden county in the US. These analyses are subject to some limitations. First, we had access to variables from the RVCT and the HPI geographic variable, but we did not have more detailed information from a chart review. For example, while diabetes was associated with APT, we do not know the severity or level of control of diabetes among cases. Moreover, factors such as homelessness and drug use were based on self-report and may be underestimated. Second, our data are limited to one county and may reduce generalizability, though it is a diverse setting with a large non-US-born population and higher TB-burden. Third, while we looked at a period of ten years to increase power for detection of associations between APT, there are still instances where there were too few data to have stable estimates for measures of association, including PLWH.

## 5 Conclusion

In a high TB-burden county in the US, we noted that APT has remained stable over the last ten years and continues to represent a sizeable proportion of all pulmonary TB cases. Public health screening programs have largely focused on non-US-born and immunosuppressed individuals as those at greatest risk of TB disease [31]. However, our findings suggest that other groups, including those living with diabetes and recent drug use, may require greater recognition and efforts to improve access to care. Differences by US nativity further highlight that programs and communications may need to be tailored to the different cultural and linguistic backgrounds of these groups to be most effective. Through these efforts, the goal is to support earlier TB diagnosis and treatment in order to reduce the morbidity and mortality of severe TB disease and prevent ongoing community transmission.

## Ethical statement

This analysis and collection of TB surveillance data were performed as part of ACPHD’s public health activities and purposes to conduct surveillance, assess, and inform local public health interventions; thus no human subject review was required in accordance with U.S. Code of Federal Regulations, 45 CFR 46.101.

## CRediT authorship contribution statement

**Rachel Marusinec:** Conceptualization, Formal analysis, Writing – original draft, Writing – review & editing. **Tessa Clifton:** Conceptualization, Formal analysis, Writing – original draft, Writing – review & editing. **Amit S. Chitnis:** Conceptualization, Writing – review & editing. **Devan Jaganath:** Conceptualization, Writing – review & editing.

## Declaration of competing interest

The authors declare that they have no known competing financial interests or personal relationships that could have appeared to influence the work reported in this paper.
